# Dr. Smith on Fever

**Published:** 1830-07-01

**Authors:** 


### XXIX.

### Dr. Smith on Fever.

In a long course of Journalism we do not remember to have received more than half a dozen formal "reclamations" from the various authors whose works have been reviewed, although we freely opened an extra limites department for that especial purpose. We believe that few journalists could make the same remark; and we take some credit to ourselves for having steered a course so generally free from recrimination. We willingly give insertion to the following letter from Dr. Smith, and shall not accompany it by any comments.

To THE EDITOR OF THE MEDICO-CHIRURGICAL REVIEW.

*Dear Sir,*

Perhaps you will allow me to correct a mistake into which you have inadvertently fallen in the concluding part of your review of my work on Fever, in commenting on the case of Dr. Dill. The mistake relates to the period of the disease at which you were called to see our mutual friend. You did not, if you remember, visit him "in the midst of the active discipline" to which he was subjected, but at the close of it; the case at the period you saw him, as you intimate, admitted of no further depletion, and no more blood was taken after that time, until he relapsed at Brighton. It is scarcely necessary to add, that the statement at the foot-note of my Treatise of the total quantity of blood abstracted (120 pounds) is a misprint for 120 ounces.

The detail of the early symptoms of this case is given at page 109 of the Treatise, while the account of the treatment is postponed to page 398, and, for the sake of brevity, the symptoms were not again recounted; but I must have been wrong in separating, at so great a distance, the symptoms from the treatment, since it has been the means of leading so acute a reader into a misconception of the stage of the disease to which the active treatment was confined.

The same reason, I hope, will account for your having thought that I could scarcely foresee, at the period at which I first abstracted blood, the severe fever that was at hand: but, if you will take the trouble to turn to page 109, you will perceive that, on the afternoon of the first day of the disease, there was present an assemblage of symptoms which clearly indicated the approach of a high degree of cerebral affection, and that accordingly, on the morning of the second day, the symptoms of intense disease of the brain were urgent: these, though occasionally mitigated, remained unsubdued through the third and the fourth days, and required the copious depletion that was resorted to; but, on the evening of the fourth or the morning of the fifth day, when you first saw Dr. Dill, they were overcome, and from that period no active remedy, excepting the cold dash, was employed. The patient was convalescent and sitting up in bed on the 8th or 9th day of the disease, a most remarkable occurrence after such an attack, and one which certainly would not have been witnessed had the malady been allowed to produce organic change in the brain, or had the means employed to prevent this result been pushed either beyond the strength of the disease or that of the patient.

Permit me to add, that this form of fever is not, I apprehend, generally understood; and yet the life of the patient depends upon its being discriminated at the very onset of the attack; the postponement of the proper remedies, for the space of a few hours, will make all the difference between life and death. In the commencement of fever, a slow and oppressed pulse, or a slow and intermittent pulse, accompanied with suspirious respiration, or respiration interrupted with frequent sighing, denotes one form of the acutest cerebral disease ever witnessed in the fevers of this country. If, in such cases, copious bleeding be not employed on the first or second day, the patient will generally be in a state of hopeless typhus on the fourth or fifth. In the convalescent stage of fever, on the contrary, a slow and intermittent pulse affords the most favorable omen: and it is one of frequent occurrence. As I had not seen it noticed in any book, nor heard it spoken of by any physician, I was at first not without apprehension for the fate of the patients in whom it occurred, although I could perceive no unfavorable symptom accompanying it. But I soon observed that it was the sure sign of a safe and steady convalescence. Of all the relapses that I have witnessed, I do not remember one in which this state of the pulse existed. It is not a little remarkable, that the same external sign thus indicates two such opposite states of the system, as that present at the commencement and at the termination of fever, and should afford two such opposite prognostics. I have to regret that my endeavour to enable my readers to distinguish and to contrast these opposite states, in order to prevent groundless apprehension in the last case, and to create immediate alarm in the first, has been attended, in one instance at least, with little success; for one of your contemporaries, after citing my statement, that at the termination of fever a slow and intermittent pulse is a sign of a sure and steady convalescence, observes —"we must pause here, and remark that an intermittent and slow pulse has been long looked on as a very unfavorable symptom in fever, and so the author admits in a subsequent page—an oversight for which we cannot account."*

Out of the last 300 patients admitted into the London Fever Hospital, I have examined the number not bled, the number bled, the number of ounces of blood abstracted from the whole, the mean quantity taken from each, and the duration of the fever and the event in both sets of cases: the result is curious and instructive. Out of these 300 patients 158 were not bled; 11 were cupped; 131 were bled from the arm; while, in all, leeches were occasionally applied, but these have not been taken into the account. Of the 158 that were not bled there died 38, or one in four. Of those who recovered, there were dismissed from the hospital, after having been under treatment in the house one week, 21—two weeks, 50—3 weeks, 38, and four weeks and longer 49. From the 11 that were cupped, the total quantity of blood abstracted was 138 ounces or 12½ ounces for each; of these patients five died, or nearly one half. From the 131 that were bled from the arm, the total quantity of blood taken was 2557 ounces, or from 19 to 20 ounces from each: of these there occurred 12 deaths, or one in 11 nearly. Of those that recovered, there were dismissed from the hospital in the first week 12—in the second week 40—in the third week, 41—in the fourth week or later 38.

The proportion of deaths in the list of those not bled, and of those cupped, is without doubt increased, by its containing those who were received into the hospital at too late a period of the disease to admit of general bleeding; but, on the other hand, the proportion of recoveries, and the rapidity of recovery, are augmented by its containing those in whom the disease was so mild, as to require neither bleeding nor any active remedy; while the 131 that were bled offered a fair average of the state of fever, as it occurs for treatment to the physician in ordinary practice, but not as it occurs to the general practitioner, who, in consequence of seeing his patients at a much earlier period of the disease than the physician, can abstract blood with incomparably greater advantage. Were this truth duly impressed upon the mind of this part of the profession, and could they be induced, during the first, second, and third days of the disease, to use the lancet with cautious boldness until the pain of the head is subdued—the mortality of fever would be diminished at least one half, and its duration a week or a fortnight.

I am, Sir,
Yours, very truly,
SOUTHWOOD SMITH.

36, *New Broad-st.*

*May*, 1830.

* London Med. and Surg. Journal, Feb. page 114.

